# Sinusoidal Vibration Source Localization in Two-Dimensional Space Around the Hand

**DOI:** 10.3389/fpsyg.2022.878397

**Published:** 2022-06-10

**Authors:** Yusuke Ujitoko, Scinob Kuroki

**Affiliations:** NTT Communication Science Laboratories, Nippon Telegraph and Telephone Corporation, Atsugi, Japan

**Keywords:** vibrotactile, haptics, localization, sinusoidal signal, user interface, direction recognition

## Abstract

There are use cases where presenting spatial information *via* the tactile sense is useful (e.g., situations where visual and audio senses are not available). Conventional methods that directly attach a vibrotactile array to a user's body present spatial information such as direction by having users localize the vibration source from among the sources in the array. These methods suffer from problems such as heat generation of the actuator or the installation cost of the actuators in a limited space. A promising method of coping with these problems is to place the vibrotactile array at a distance from the body, instead of directly attaching it to the body, with the aim of presenting spatial information in the same way as the conventional method. The present study investigates the method's effectiveness by means of a psychophysical experiment. Specifically, we presented users with sinusoidal vibrations from remote vibrotactile arrays in the space around the hand and asked them to localize the source of the vibration. We conducted an experiment to investigate the localization ability by using two vibration frequencies (30 Hz as a low frequency and 230 Hz as a high frequency). We chose these two frequencies since they effectively activate two distinctive vibrotactile channels: the rapidly adapting afferent channel and the Pacinian channel. The experimental results showed that humans can recognize the direction of the vibration source, but not the distance, regardless of the source frequency. The accuracy of the direction recognition varied slightly according to the vibration source direction, and also according to the vibration frequency. This suggests that the calibration of stimulus direction is required in the case of both high and low frequencies for presenting direction accurately as intended. In addition, the accuracy variance of direction recognition increased as the source became farther away, and the degree of increase was especially large with the low-frequency source. This suggests that a high frequency is recommended for presenting accurate direction with low variance.

## 1. Introduction

There are situations where it is beneficial to present spatial information to the user, such as informing the driver of the direction and distance of other cars and obstacles around the car. Since the tactile sense is often more available than the visual and auditory senses while driving, it would be desirable if it were possible to present information to the driver through the tactile sense (Scott and Gray, 2008). Obstacle detection systems have already been implemented in vehicles to alert the driver of approaching hazards within a certain distance with a warning vibration (Cadillac, 2012), but it would be more useful if the system could present spatial information such as “in which direction” and “at what distance” the hazards were located. Of course, such a method (using tactile sensation to present spatial information) is not limited to use cases related to cars but would also be effective in situations where other audio-visual channels are overloaded, and in use cases required by people with audio-visual disabilities.

In this study, we focus on vibrotactile stimulation, which can be implemented inexpensively, rather than other kinds of tactile stimuli (Choi and Kuchenbecker, 2012). The vibrotactile stimuli are already familiar to us as they are built into common touch panels and game controllers. There have been several studies that have used these touch panels and game controllers to present spatio-temporal information by means of vibration (Lee and Choi, 2013), but most of them examined this in situations with a clear context, such as presenting vibrations along with visual cues to represent a ball rolling around in a box. In contrast to these previous studies, we focus on the possibility of presenting spatial information with only tactile cues.

In previous studies that investigated the use of vibration to present spatial information to a user, the method adopted was to directly place a vibrotactile array in contact with a part of the user's body [e.g., hand (Günther et al., 2018), wrist (Chen et al., 2008), or torso (van Erp, 2008)]. With this approach, users can identify the position of the vibration source by detecting the position of the most vibrating skin since there is no distance between the vibration array and the skin. Although this approach is straightforward, it faces some issues. One is the problem of laying out the vibrotactile array in a limited space directly contacting the body. If we want to present direction with a high resolution, we need to arrange the dense vibrotactile array, but it is expensive to miniaturize and integrate an array in a limited space. Another issue is the problem of heat generation. The heat generated by the presentation system including the vibrotactile array can make it uncomfortable to use and may disturb the tactile sensation (the heat problem was mentioned by Wentink et al., 2011; Jeon et al., 2013). These problems are unavoidable when the vibrotactile array is in contact with the body.

In our previous study, we proposed a method of presenting impact vibrations to the user's body from a remotely distributed vibrotactile array through a medium (Ujitoko et al., 2021). Since this method does not require the array to directly contact the body, it alleviates the problem of laying out the actuators in a limited space and the problem of heat generation. With this method, the user needs to recognize the direction and distance of a remote vibration source based on the propagated vibration. We have investigated the accuracy of source localization with an impact vibration, and found that the direction could be recognized to some extent, but the distance could not be recognized accurately (Ujitoko et al., 2021). Based on the same idea as that proposed in our previous study (Ujitoko et al., 2021), the present study investigates the accuracy of source localization but this time using a sinusoidal vibration due to following reasons. First, voice-coil motors, which are usually used to simulate sinusoidal vibrations, are a better option for tactile presentation than solenoids, which are usually used to simulate impulse vibrations, in the following three ways: power consumption (see Immersion, 2020), heat, and controllability (see Choi and Kuchenbecker, 2012). Indeed, ordinary mobile phones such as iPhone and game controllers such as Nintendo switch controller adopt voice-coil motors instead of solenoids. Second, the presentation of sinusoidal vibrations can allow control of the quality of presented vibrotactile information by manipulating the vibration frequency. For example, let us consider the use case when the users need to recognize and discriminate between obstacles with different object characteristics (e.g., cars and pedestrians) that are present in different locations. The requirements for such cases would be realized by representing the spatial information of different objects with different frequencies of sinusoidal vibrations. It is known that humans are good at discriminating the frequency of vibrations, and that the texture rendered by low-frequency vibrations and that rendered by high-frequency vibrations are perceived to be different (Goff, 1967; Kuroki et al., 2013).

In this study, as the first step, we conducted experiments to determine how well distance and direction can be recognized when high frequency (230 Hz) and low frequency (30 Hz) sinusoidal vibrations are applied. We chose these two frequencies since they effectively activate two distinctive vibrotactile channels: the rapidly adapting afferent channel and the Pacinian channel (Johansson et al., 1982). Based on the experimental results, we compared the results between sinusoidal vibration and impulse vibration, and between the different stimulus frequencies of sinusoidal vibrations. One of the contributions of the results of this study is that the use of sinusoidal vibration succeeded in reproducing the same results as impulse vibration, where direction could be recognized to some extent, but the distance could not be recognized accurately. In addition, our results showed the frequency-dependent effect on the accuracy of direction recognition. Based on these results, we discussed how sinusoidal vibrations should be used for spatial information presentation in applications.

## 2. Related Work

In this section, we introduce previous studies in which the vibration localization was investigated. We divided these studies into two categories: those in which the vibrotactile actuators were placed on the body surface and those in which they are placed away from the body.

### 2.1. Placing Vibrotactile Actuators on the Body Surface

The localization accuracy of vibration sources has been investigated by placing multiple vibrotactile actuators on various parts of the body surface. Specific body parts that have been investigated include the hand (Elvitigala et al., 2019), wrist (Chen et al., 2008; Lee et al., 2015), arm (Cholewiak and Collins, 2003; Oakley et al., 2006), abdomen (Cholewiak et al., 2004; Cholewiak and McGrath, 2006; van Erp, 2008), waist (Jones and Ray, 2008), back (Lindeman and Yanagida, 2003), and head (Diener et al., 2017). For example, in the study by Chen et al. (2008) a 3 × 3 vibrotactile actuator was placed on the wrist. Similarly, the study by Sofia and Jones (2013) also placed 3 × 3 vibrotactile actuators on the palm. The localization ability depended not only on the body part, but also on the number of vibrotactile actuators and the density of the actuator locations. In the study by Cholewiak et al. (2004), for example, the localization accuracy ranged from 97% (when the number of vibrotactile actuators was 6 on the abdomen, and the distance between vibrating actuators was 140 mm) to 74% (when the number of vibrotactile actuators was 12 on the abdomen, and the distance between vibrating actuators was 72 mm).

These studies examined the localization accuracy on the surface of the body where the source actuator was actually placed, while there are also studies that attempted to virtually localize the source outside the body by using illusions. Phantom sensation, which is also known as tactile funneling, is a phenomenon in which a source vibration that does not actually exist is illusorily localized on the body surface between actuators that present the vibrations (Bekesy, 1967). By presenting stimuli with actuators in each hand, some researchers extended the phantom sensation to attempt to localize the source in the space between (Patel et al., 2019) and outside (Tawa et al., 2020) both hands.

The methods of research described in this section have the potential to present spatial information, but as mentioned in the Introduction, placing the vibrotactile actuators on the body surface may cause layout and heat generation problems, so in this research we investigate the method of placing the vibrotactile actuators outside the body.

### 2.2. Placing Vibrotactile Actuators Outside the Body

The localization accuracy of vibration sources has also been investigated when the actuators were placed on the tool being held by the subject. Miller et al. (2018, 2019) had blind-folded subjects hold one end of an one-dimensional medium (such as a stick) and actively shake it with their hands, to determine if they could estimate the point of contact when the medium hit an object. Sreng et al. (2008) had subjects hold one end of a stick and used several vibration patterns to estimate the vibration source location. In these studies (Sreng et al., 2008; Miller et al., 2018, 2019), the subjects held a one-dimensional medium, so the vibration source localization is equivalent to distance estimation.

On the other hand, in our previous study, we examined the localization ability of a hand placed on a two-dimensional medium when some place on the medium around the hand was presented with an impact vibration (Ujitoko et al., 2021). If the subjects can localize the vibration source, it will be possible to present not only distance but also directional information. We found that the subjects could recognize direction to some extent, but not distance, when the source was an impact vibration. We also tested the localization ability with different hand posture conditions, and found that the localization performance was better when the whole hand (five fingers and the palm) was grounded than when only five fingertips were grounded. Still, it remains unknown to what extent direction and distance recognition becomes possible with sinusoidal vibration.

In this study, we investigated the localization performance when using low- or high-frequency vibrations (30 Hz as low frequency and 230 Hz as high frequency) as a signal source, under similar experimental conditions to those used in our previous study (Ujitoko et al., 2021), including the layout of stimulus sources and the medium material. For the hand posture condition, we chose the condition where the whole hand was grounded on the medium, since the localization accuracy was higher with this posture than when only the five fingertips were grounded (Ujitoko et al., 2021).

## 3. Experiment

### 3.1. Subjects

Ten subjects [two males and eight females, all right-handed, with a mean age of 36.5 (SD: 9.0) years] participated. The mean length and width of subjects' hands were 16.47 (SD: 0.73) cm and 15.53 (SD: 1.13) cm, respectively with fingers extended. The mean hand size which corresponds to the mean value of height and width was 16.00 (SD: 1.06) cm. The mean hand size of two males was 15.75 (SD: 1.06) cm and that of eight females was 16.06 (SD: 0.69) cm, with negligible differences depending on gender. All subjects were ignorant of the purpose of the study. Ethical approval for this study was obtained from the ethics committee at Nippon Telegraph and Telephone Corporation (Approval number: R02-015 by NTT Communication Science Laboratories Ethics Committee). The experiments were conducted according to the principles that have their origin in the Helsinki Declaration. Written informed consent was obtained from all subjects in this study.

### 3.2. Apparatus and Stimulus

**Figure 1:**
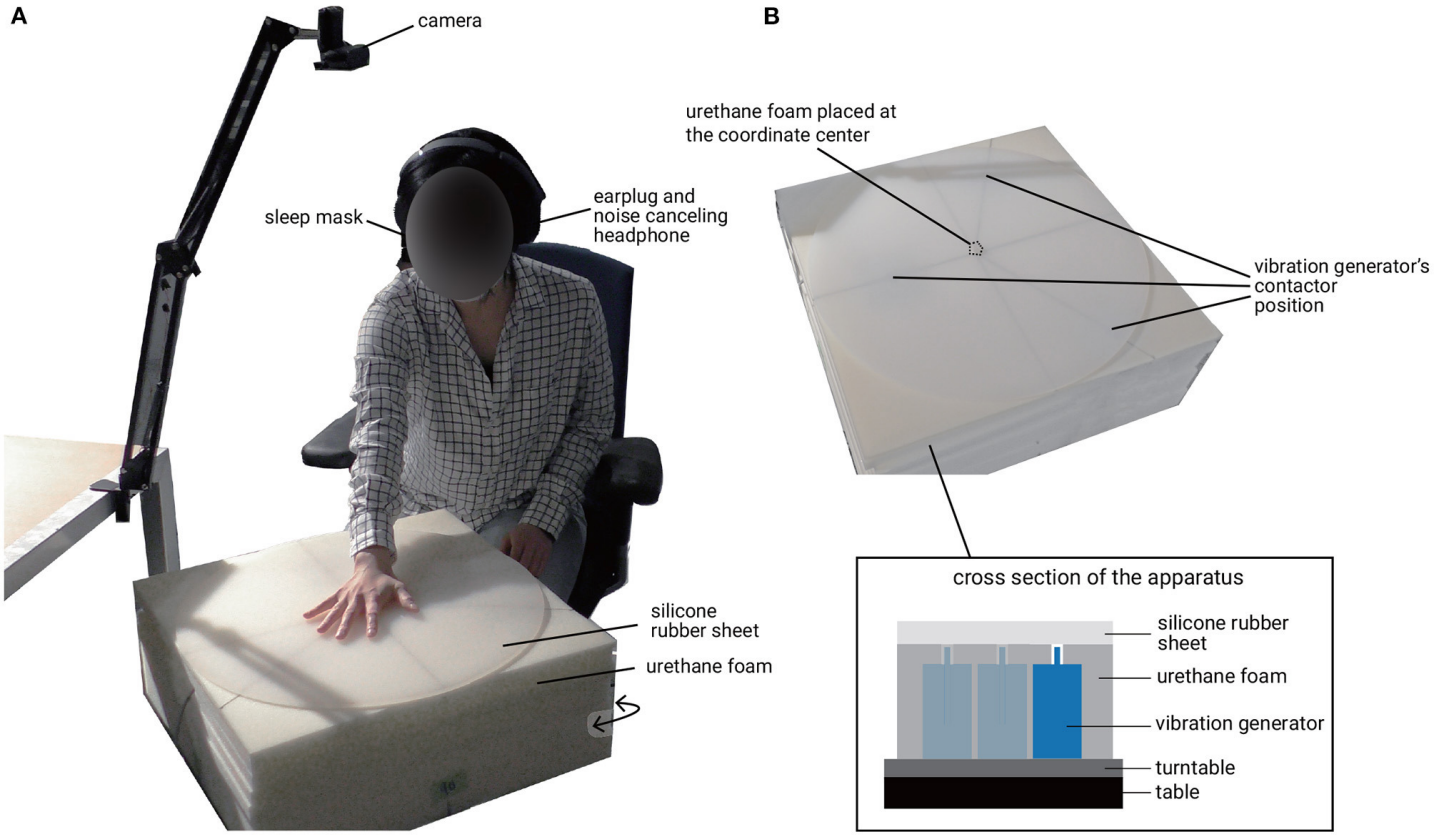
Experimental environment. **(A)** Overview. **(B)** Apparatus.

Figure 1 shows the experimental environment. Subjects were seated comfortably on a chair. To prevent the subjects from receiving visual information, such as a change in the reflection of the sheet's illumination when a location under the sheet was stimulated, the subjects were blindfolded during the task with a sleep mask. They wore earplugs and noise-canceling headphones playing white noise to muffle external sounds. They put their right hand on the silicone rubber sheet and adjusted the center of the hand to the center of the silicone rubber sheet. A small piece of urethane foam was placed at the center of the silicone rubber sheet as a cue for the adjustment of the hand placement. The silicone rubber sheet was circular, and the radius was 250 mm. The thickness of the sheet was 5 mm. The hardness of the silicone rubber sheet was 30 (tested with a durometer of type Shore A). To compare the result with our previous study (Ujitoko et al., 2021) which presented impulse vibration on a silicone medium, we used the same silicone rubber sheet.

A green sticker was placed on the nail of the index finger of the subject's left hand. Subjects indicated the position where they thought the vibration source was by pointing with their left hand index finger to the position on the silicone rubber sheet. A camera above the silicone rubber sheet took pictures and identified the green sticker's position, which represented the position indicated.

**Figure 2:**
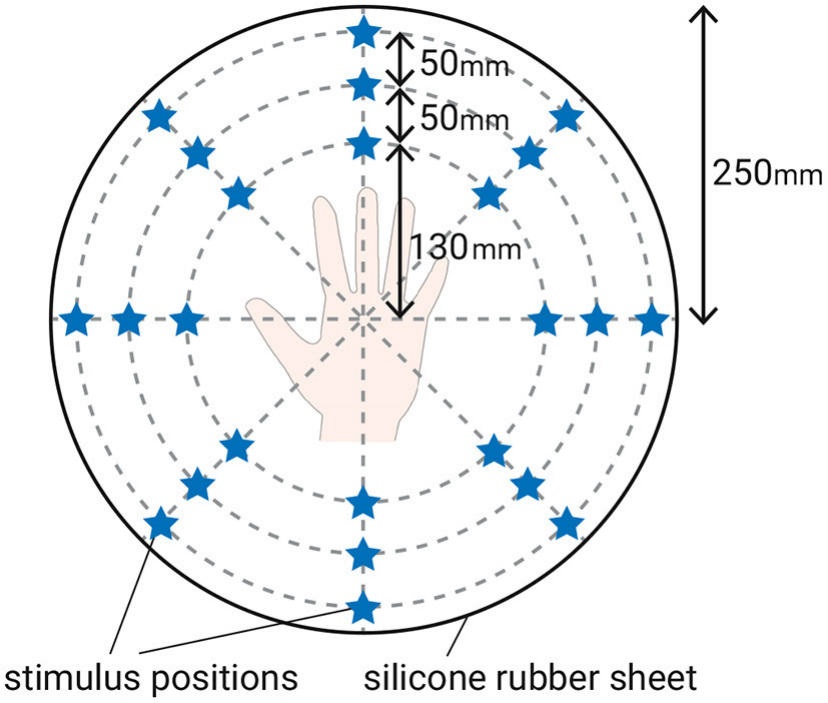
Layout of stimuli. The star-shaped marker indicates the position of the stimulus.

Twenty-four points were stimulated by the vibration generators in eight directions at three stimulus distances (shown in Figure 2). The layout of stimuli was the same as our previous study (Ujitoko et al., 2021), which enabled the comparison with the previous study. The diameters of stimuli from the center were 130, 180, and 230 mm. There were eight stimulus directions from the center. To generate the sinusoidal vibrations, we used specific vibration generators (Emic corporation, 511-A) that have been used in our past research (Watanabe et al., 2010; Kuroki et al., 2012; Kuroki and Nishida, 2018). The vibration generators could control the frequency and amplitude independently. The size of the vibration generators was 120 mm wide, 100 mm deep, and 190 mm high. They were too large to arrange them in the layout shown in Figure 2. Thus, we placed only three vibration generators for each of three stimulus diameters on the turntable and by rotating the turntable, 24 points could be stimulated. In order to ensure that the vibration generator made a good contact with the silicone rubber sheet, the gap was filled with urethane foam. The diameters of the vibration generators' contactors were 6 mm. Subjects did not know that only those discrete 24 points would be stimulated. Subjects only knew that some location on the whole area of the silicon rubber sheet would be stimulated.

We chose 30 and 230 Hz vibrations as source signals; these were selected to effectively activate two distinctive vibrotactile channels: the rapidly adapting (RA) afferent channel that is sensitive to lower vibration frequencies and the Pacinian (PC) channel that is sensitive to higher frequencies. Regarding the duration of vibration, we consider that a shorter duration is preferable for immediate presentation of spatial information with vibration in applications. In addition, our previous report showed that humans discriminated the vibration frequencies with 0.2 s duration of vibrations (Kuroki et al., 2013). Thus, we configured the duration of the vibration to 0.2 s.

In advance of the experiment, the amplitudes of both vibrations sources were configured so that the perceptual intensities were matched. The human's perceptual detection threshold depends on the vibration frequency (Bolanowski et al., 1988). Due to this perceptual characteristic, even when the vibration amplitudes are physically the same, the perceived intensity of the vibrations would be different when the vibration frequencies are different. In our experiment, the perceived intensity was matched to examine the effect of difference in frequency, rather than that in intensity, on localization ability. We set the acceleration sensor (MPU-6050) on the silicone rubber sheet at the point of the contactor and measured the maximum amplitude of acceleration. The maximum amplitudes were 1.97 G at 30 Hz and 8.92 G at 230 Hz.

Also, we measured propagated vibration at the point of the center of the medium. When the vibration frequency was 30 Hz, we observed the propagated vibration with a maximum frequency component of 30 Hz and its harmonic components (60 Hz). We also found some attenuation due to propagation.

### 3.3. Procedure

This experiment used a within-subject design. At the beginning of the experiment, the subjects were presented with written instructions that described the situation and procedures of the experiment. After reading this, subjects moved on to the experiment.

The experiment was composed of a familiarization phase and a test phase (see the flow of experiment in Supplementary Figure 1). In the familiarization phase, there were six trials. The procedure of the trials in the familiarization phase was the same as that in the test phase. The six points of stimulation out of 24 points assigned to the six trials were selected randomly. The frequency of the vibration used for stimulation in the familiarization phase was the same as in the first block of the test phase.

In the test phase, there were 12 blocks. Each block was composed of 24 trials. Thus, the total number of trials was 288 per subject. Each of the 24 points was stimulated in a separate trial in the block. The order of the 24 points was pseudo-randomly assigned. In any one block, the frequency of the vibration was fixed at 30 or 230 Hz. The 30 and 230 Hz were assigned alternately to blocks. The assignment of 30 or 230 Hz to the first block in the test phase was balanced across the subjects.

At the start of every trial, the experimenter rotated the turntable to adjust the position of the vibration generator to the designated position and rang a bell. Even when subjects wore earplugs and noise-canceling headphones with white noise, they could hear the bell ringing. Then, subjects put their right hand on the silicone rubber sheet by identifying the small piece of urethane foam positioned at the center of the sheet. Next, one of the 24 points was stimulated. Subjects then withdrew their right hand and pointed their left hand index finger to the point where the subjects speculated the point of stimulation to be. After recording by the camera to identify the point of the subject's left index finger, the experimenter rang a bell again. The subjects then withdrew their left index finger from the sheet.

After all trials in a block had been completed, there was at least a 10 min break before the next block started.

## 4. Results and Discussions

### 4.1. Overall Results

**Figure 3:**
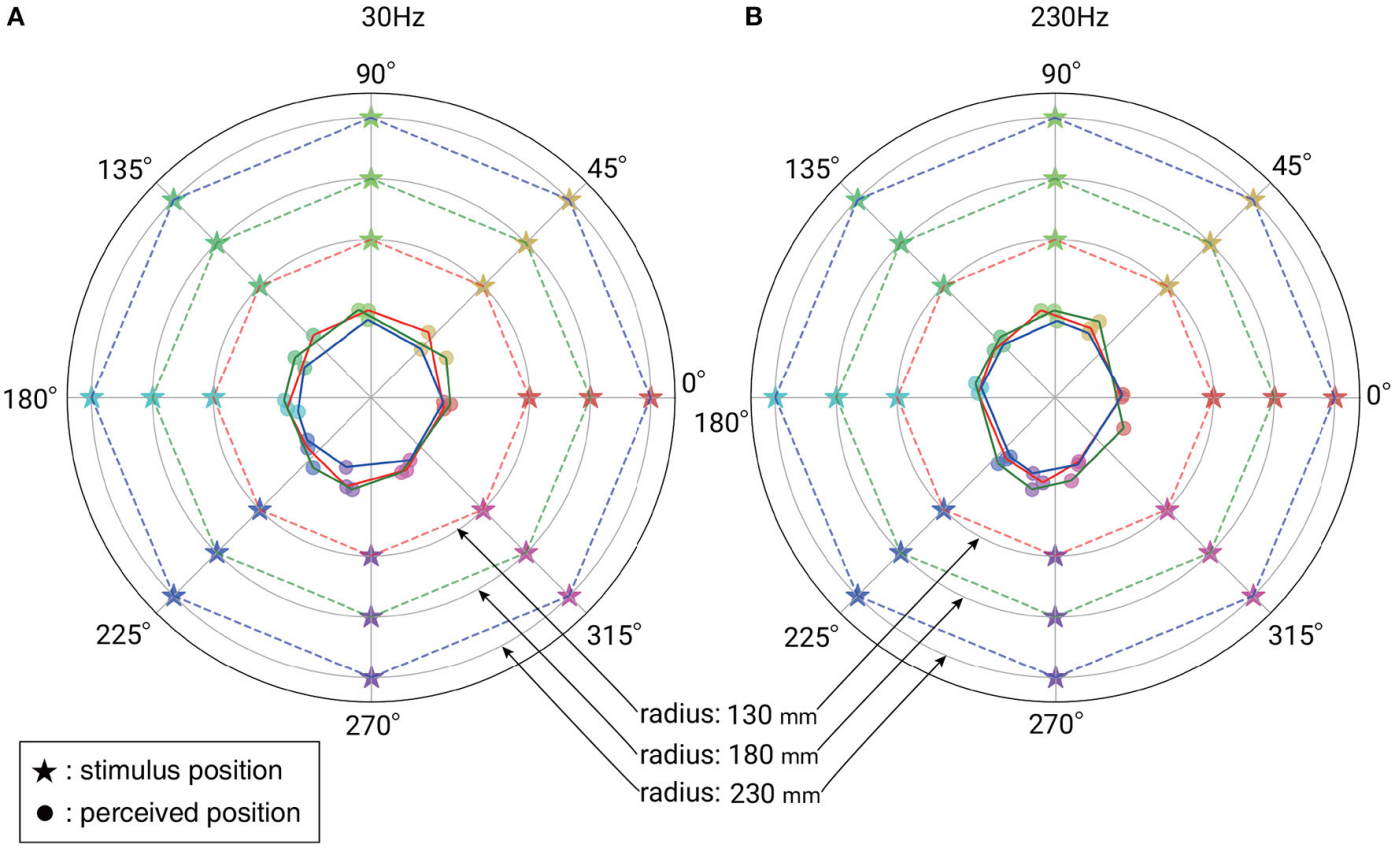
**(A)** Mean perceived positions for each stimulus in the case of 30 Hz vibration. **(B)** Mean perceived positions in the case of 230 Hz vibration. A star-shaped marker indicates the stimulus position. A circular marker indicates the perceived position for each stimulus position. The dashed lines and solid lines are visual aids representing the stimulus space and the perceptual space. Use of the same color for the dashed line and solid line means the corresponding relationship of stimulus and perception.

**Figure 4:**
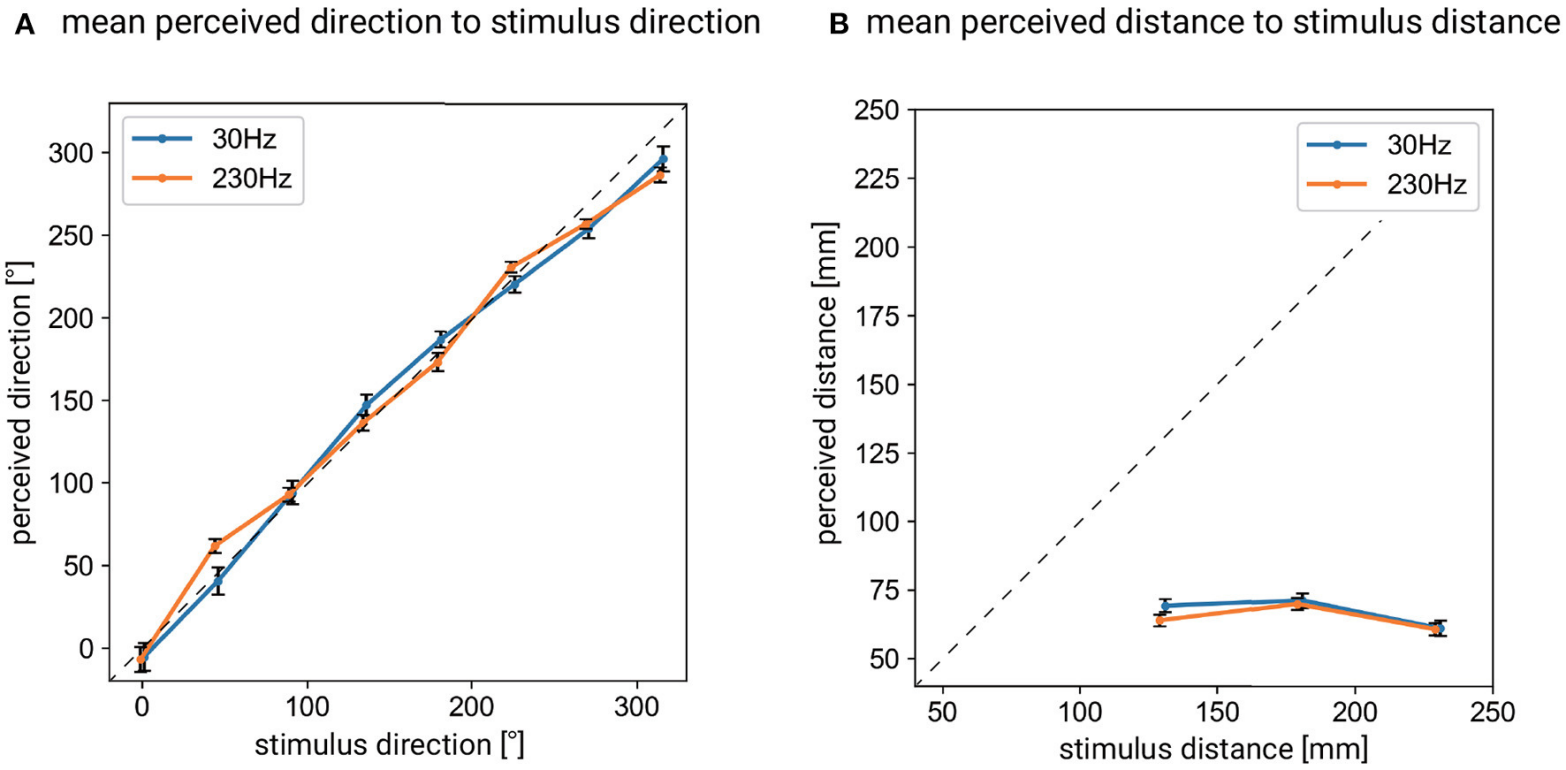
**(A)** Mean perceived direction for each stimulus direction in the cases of 30 and 230 Hz vibrations. **(B)** Mean perceived distance for each stimulus distance in the cases of 30 and 230 Hz vibrations. Error bars denote 95%CI.

Figures 3A,B show the mean perceived position for each stimulus position at 30 and 230 Hz, respectively. Figure 4A shows the mean perceived direction for each stimulus direction and Figure 4B shows the mean perceived distance for each stimulus distance. The perceived direction was calculated as the distance between the point where subject pointed and the center of the hand. Both of these Figures 3, 4B suggest that subjects had difficulty in identifying the distance of the vibration source accurately under both frequency conditions. Since the mean size of the subjects' hands was 160.0 (SD: 10.6) mm and the mean perceived distance was <75.0 mm, this shows that the mean perceived distance was inside the subject's hand size. This indicates that the vibration source outside of the hand was localized inside the area of the hand.

On the other hand, Figure 4A suggests that the subjects could recognize the direction of the vibration source. In summary, the method of localizing a sinusoidal vibration source around the hand is not promising as a distance presentation method, but is promising as a direction presentation method. In the following sections, we discuss the results of the analysis on direction recognition in more detail.

### 4.2. Results of Direction Recognition

In this analysis, we focus on the directional bias and the standard deviation of the directional bias. The directional bias here is also called “constant error” or “systematic error,” and the bias is calculated as the difference between the stimulus direction and the perceived direction. The bias and the standard deviation of the bias are metrics that have been systematically investigated in the series of studies on vibration localization (Cholewiak et al., 2004; van Erp, 2008; Kappers et al., 2020). From the viewpoint of applications presenting spatial information, the bias is a measure of whether the intended direction is perceived by the user or not. The standard deviation of the bias is related to the stability of the perceived direction. Therefore, both of them are important metrics from the viewpoint of application.

#### 4.2.1. Directional Bias

For the bias, we conducted two tests: (1) a test to determine whether there was a significant difference in bias between different vibration frequency conditions, and (2) a test to determine under which stimulus conditions there was a significant difference between the presented and perceived position of the stimulus (and hence to determine under which stimulus conditions the bias was not statistically significant).

**Figure 5:**
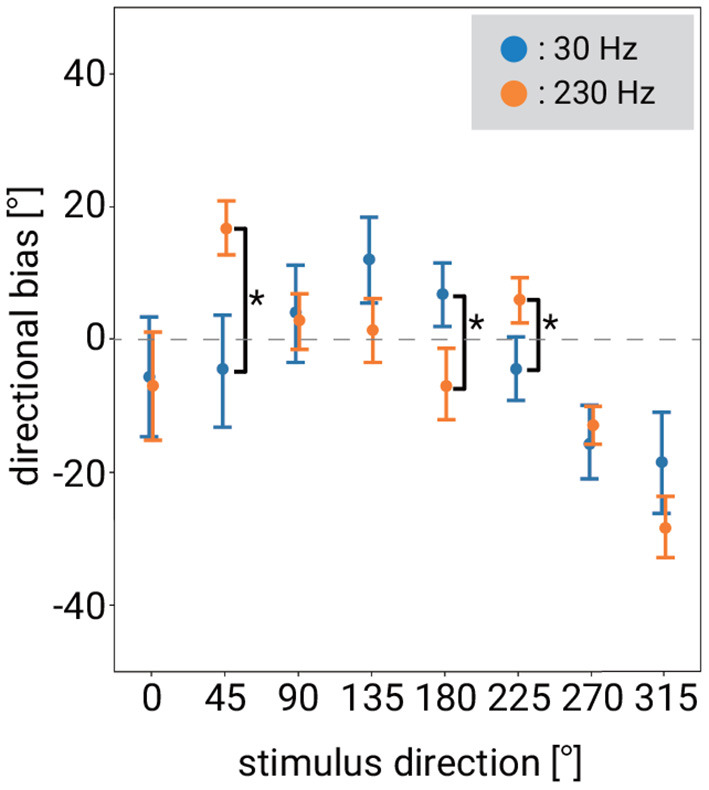
Difference of mean directional biases for each stimulus direction between 30 and 230 Hz. Error bars denote 95%CI. The asterisk indicates significant difference between stimulus frequency.

Regarding test (1), firstly, in order to clarify how to merge the data to compare the bias between frequencies, we conducted an analysis of variance (ANOVA) on three factors: frequency, stimulus distance, and stimulus direction. Since the Shapiro-Wilk test showed the lack of normality of our data, we applied Aligned Rank Transform (ART) (Wobbrock et al., 2011) and then conducted the ANOVA. Full results of the ANOVA can be found in Supplementary Table 1. As a result, since there was a significant interaction effect between frequency and stimulus direction, we performed a *post-hoc* comparison between frequencies for each stimulus direction using ART applied data as an analysis for (1). In contrast, since there was no significant interaction between frequency and stimulus distance or among the three factors, we did not examine the *post-hoc* test in terms of stimulus distance. The results are shown in Figure 5. There was a difference between 30 and 230 Hz at 45, 180, and 225°.

**Figure 6:**
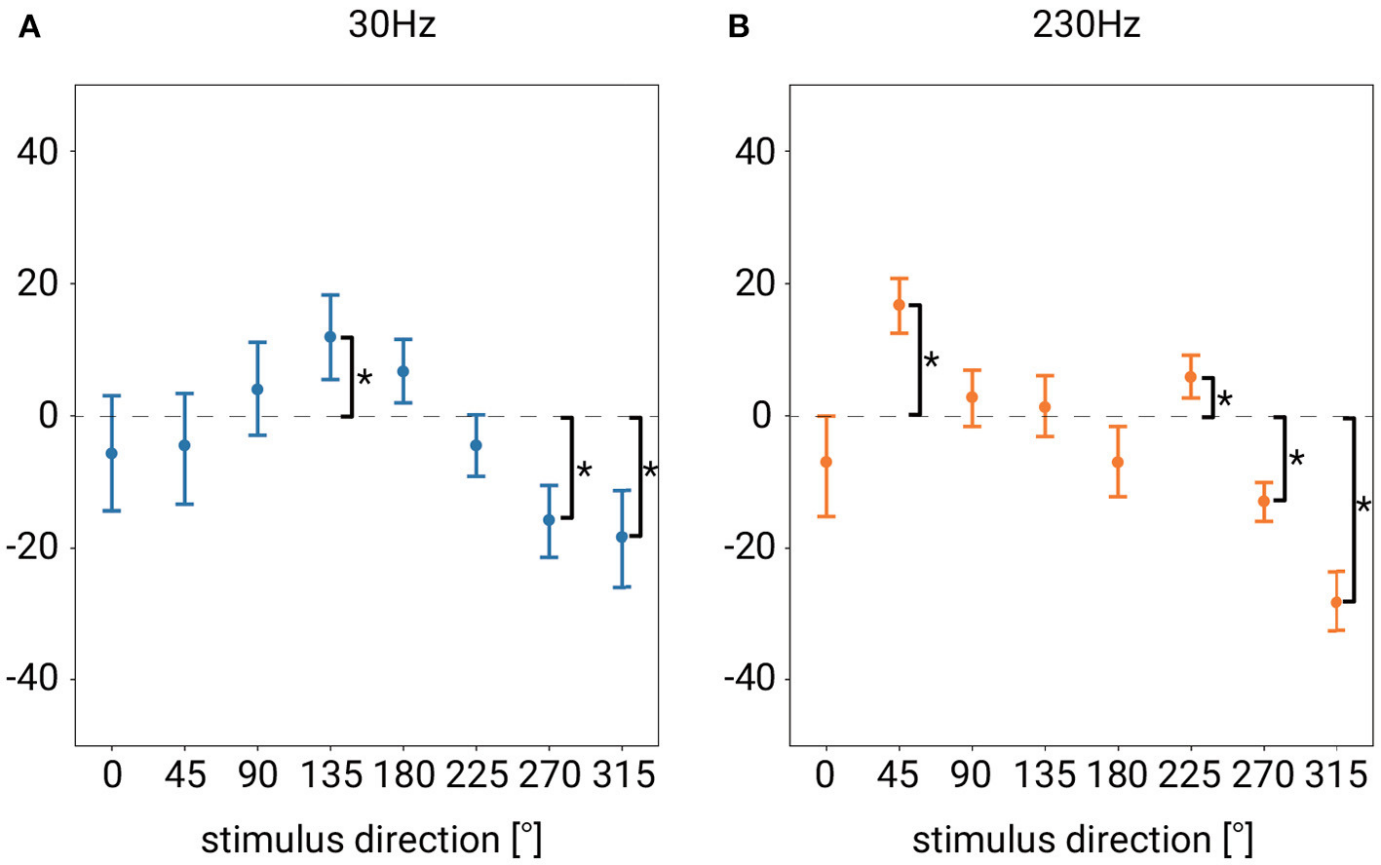
Re-plot of Figure 5, for each vibration frequency of the stimulus **(A)** Mean directional bias for stimulus direction at 30 Hz. **(B)** Mean directional bias for stimulus direction at 230 Hz. Error bars denote 95%CI. The asterisk indicates significant difference from 0.

Regarding test (2), we tested under which stimulus conditions there was a significant difference between the presented and perceived position of the stimulus (and hence, under which stimulus conditions the directional bias was not significant). According to the results of the ANOVA described above, the effect of stimulus distance on bias was not significant, and thus we did not test in terms of stimulus distance. We tested for the combined conditions of each stimulus direction and each frequency. Figure 6 is a re-plot of the data in Figure 5, with the vibration frequencies separated. To clarify whether a directional bias existed in any of the conditions, we computed 10,000 bootstrap samples of the bias (Efron and Tibshirani, 1994). If the Bonferroni-corrected 95% confidence intervals (CIs) did not overlap zero, we concluded that the bias was statistically significant.

Under almost half of the stimulus direction conditions, the direction was correctly recognized (i.e., the bias was not significant), but in some directions, the bias was significantly not zero (see Figures 6A,B). A negative bias was observed for both 30 and 230 Hz when the stimulus direction was 270 and 315°. This means that the perceived direction deviated in the clockwise direction relative to the actual stimulus direction. The positive maximum bias was observed when the stimulus direction was 135° at 30 Hz and 45° at 230 Hz, showing that the perceived direction of that stimulus deviated in the counterclockwise direction relative to the actual direction.

#### 4.2.2. Standard Deviation of Directional Bias

**Figure 7:**
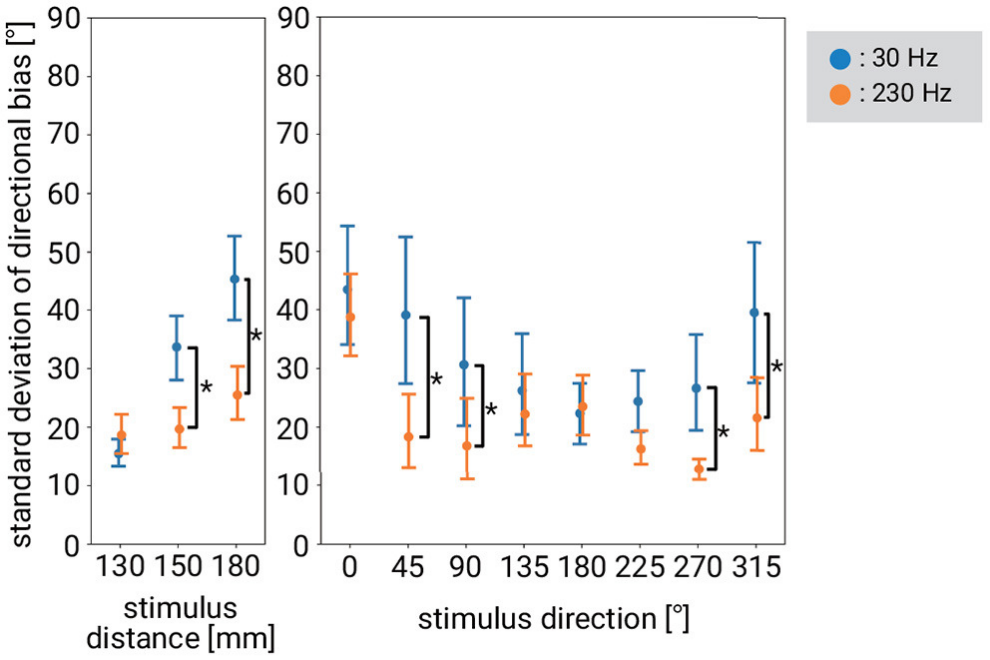
Standard deviation of directional bias for stimulus distance or stimulus direction at 30 and 230 Hz. Error bars denote 95%CI. The asterisk indicates significant difference between stimulus frequency.

The directional bias data for each subject was aggregated by stimulus distance or stimulus direction, to calculate the standard deviation. In order to clarify whether the standard deviation of the bias differed depending on the frequency, a comparison of standard deviation between frequencies was performed. Firstly, in order to clarify how to merge the data to compare the standard deviation between frequencies, we performed three-way ANOVA with factors of frequency, stimulus distance, and stimulus direction, after the application of ART to the data. Full results of ANOVA can be found in Supplementary Table 2. There was a significant interaction effect between stimulus distance and frequency, a significant interaction effect between stimulus direction and frequency, and a significant three-way interaction effect among stimulus distance, stimulus direction, and frequency. Thus, we examined whether there were differences between frequencies in (1) the standard deviation for each stimulus distance, (2) the standard deviation for each stimulus direction, and (3) the standard deviation for each combination of stimulus distance and stimulus direction. Figure 7 shows the results corresponding to (1) and (2), respectively. We conducted a *post-hoc* test to determine the stimulus conditions in which the standard deviations differed between frequencies. It was found that the standard deviation of 230 Hz was significantly smaller than that of 30 Hz when the stimulus distance was 150 and 180 mm. Also, the standard deviation of 230 Hz was significantly smaller than that of 30 Hz when the stimulus direction was 45, 90, 270, and 315°.

Regarding (3), we examined the conditions in which there was a difference in the standard deviation between frequencies in each condition (combination of stimulus direction and stimulus distance conditions). The standard deviation was significantly smaller for 230 Hz than for 30 Hz when the stimulus distance was 180 mm and the stimulus direction was 45 or 315°. There was no significant difference in the other conditions.

### 4.3. Discussions on Overall Results

Our results showed that the mean perceived position for every stimulus condition existed inside the hand area regardless of the stimulus distance, and this indicates that distance recognition was difficult for vibrations from a distant location (see Figure 4B). This gives a seemingly counter-intuitive impression, in the sense that the spatio-temporal information of the vibration propagating to the hand should change with the stimulus distance. However, within the spatial and temporal information, only the spatial intensity distribution on the skin might be used to estimate the distance, given the fact that temporal information is difficult to use as a cue in tactile sensation when localizing a vibration source (Gescheider, 1965). One possible explanation of the poor ability of distance estimation might be that the subjects lacked information about the medium (silicone rubber sheet) that mediated the vibration source and the hand, and thus subjects could not assume a model to link the intensity distribution and the stimulus distance within the limited time of the experiment without instruction. Note that the ability of direction estimation is not affected by the type of medium, since it can be recognized as the direction of the area where the perceived intensity is the strongest. The current result of the difficulty in distance recognition is consistent with the result of the experiment using impulse vibration under similar experimental conditions (Ujitoko et al., 2021). There is a difference between the current study and the previous study (Ujitoko et al., 2021) in that the average perceived position was outside the hand area in the previous study, while it was inside the hand in the current study. This may be explained by differences in the procedure. The subjects in the previous experiment (Ujitoko et al., 2021) were asked to point to the perceived position with the left hand index fingers while keeping their right hand on the medium, while the subjects in the current experiment were asked to remove their right hand and then point. The subjects in the previous study were unable to point directly to the medium under their right hand, which may have led to the assumption that the stimuli were applied from outside the hand. There are some studies that have examined the performance of tactile distance recognition in the context of sound source localization. Richardson and Frost (1979) conducted a tactile sound localization task in which subjects were asked to estimate the location of a sound source by sensing the vibration of the index fingers of both hands. In their experiment, the sound source was located around the subjects and the vibration was driven based on the perceived sound with microphones placed beside both ears. The correct response rate was 65.4% (33.3% for a random choice from three options) when the subjects were asked to answer one of “near,” “middle,” or “far” choices for the perceived distance of the sound source. In their study, they fed back the correct answers to the subjects, which may have allowed the subjects to explicitly learn the relationship between intensity and distance. In addition, differences in the presentation sites (index fingers of both hands in their experiment), the presentation waveform (the vibrations they used covered a wide range of frequencies), and the range of stimulus direction (only within the range of 120° in front of subjects) might affect the difference in results from those obtained by us.

Our results also showed that direction recognition performance was better in the sense that it had relatively fewer errors than distance recognition. The fact that we were able to recognize the direction to some extent (mean directional error at 30 Hz: 32.8° degrees, mean directional error at 230 Hz: 25.7°) is consistent with the results of experiments using impulse vibration (Ujitoko et al., 2021, mean directional error: 20.8°) and tactile sound localization study (Gescheider, 1965, mean directional error: 14.3° for tone). As mentioned above, the direction recognition could be done simply by detecting which part of the hand's skin vibrated intensively. It can be said that direction presentation in applications is promising.

**Figure 8:**
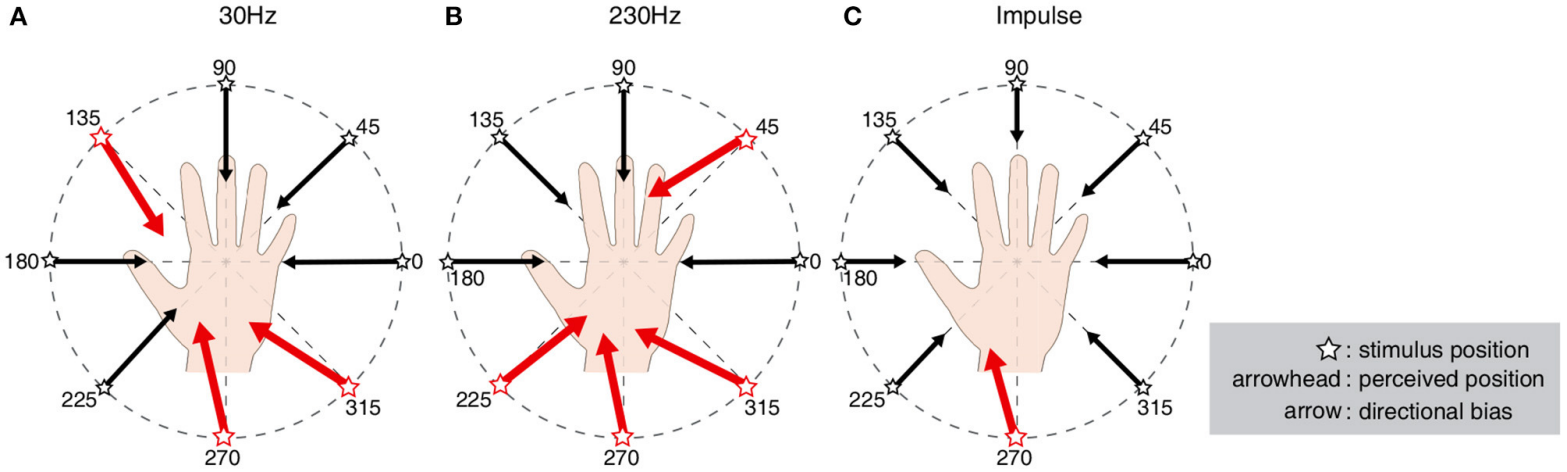
**(A)** Schematic diagram in the case of 30 Hz. **(B)** Schematic diagram in the case of 230 Hz. **(C)** Schematic diagram in the case of impulse. The Red arrows indicate that the directional bias was significant. The diagram of **(C)** was created based on the information in the paper (Ujitoko et al., 2021).

It was found that there was a significant directional bias for specific stimulus directions (Figures 8A,B shows the stimulus directions in which there was a significant bias). There was a tendency for bias when the stimulus direction was toward the base of the hand, that is the wrist. The reason why the bias was evident more on the wrist side than on the fingertip side may be related to the difference in the spatial resolution (e.g., the two-point discrimination threshold is 2 or 3 mm for the fingertip and about 10 mm for the palm) and/or in the size and layout of receptive field (Schady and Torebjörk, 1983; Vallbo and Johansson, 1984), although how this affects the results is not clear. It should be noted that, when an impulse vibration was used, with almost the same experimental setup as in the present study, the number of stimulus directions that showed a significant bias was smaller than in the present study (Ujitoko et al., 2021; see Figure 8C). The difference in the presence or absence of bias depending on the type of vibration can be attributed to various factors. One of these may be due to the difference in perceived area over which a vibration is applied between the impulse and sinusoidal vibration. Békésy (1958) investigated this point by presenting subjects with pulsed and sinusoidal cyclic vibrations and asked the subject to draw the shapes of the perceived vibration. The area drawn in the case of the pulsed vibration was smaller than that in the case of the sinusoidal vibration, even though the perceived intensity was matched. This finding is consistent with our results, which showed that fewer stimulus directions were biased by impulse vibrations than by sinusoidal vibrations. It may be also worthwhile considering a phenomenon known as phantom sensation. The phantom sensation is a phenomenon in which vibrations are localized between two distant points when two stimuli are applied simultaneously on the skin. The finding that the phantom sensation was sharply localized by impulses (clicks) rather than by cyclic vibration (Bekesy, 1967) may be related to our finding that impulse vibration localization was better than sinusoidal vibration localization in our experimental environment.

There is a previous study which is inconsistent with our result. Sherrick et al. (1990) conducted an experiment to examine the ability to distinguish between two stimulated locations on the hypothenar eminence of palm. They compared the conditions between periodic and impulse vibrations as stimuli. Their results showed that the localization acuity was worse in the case of impulse vibration than in the case of the cyclic vibration, which is inconsistent with our results. This may be because they presented the vibration directly to the skin and they covered the non-vibrating area with a board to prevent the propagation of vibration, which is different from our experimental environment.

### 4.4. Discussions on Differences in Stimulus Frequency

We found differences depending on the stimulus frequency with respect to the bias in the recognition of direction and its standard deviation. For example, at 30 Hz, there was a significant bias when stimulating from 135°, while at 230 Hz there was no significant bias when stimulating from 135°. Instead, at 230 Hz, there was significant bias when stimulating from 45 or 225°, while at 30 Hz there was no significant bias when stimulating from 45 or 225°. As for the standard deviation, the standard deviation was significantly smaller at 230 Hz than that at 30 Hz when the stimulus distance was 150 or 180 mm. There was no significant difference in the standard deviation depending on the stimulus frequency when the vibration source was closer to the hand, although, as mentioned, when the vibration source was far from the hand, the standard deviation was significantly smaller at 230 Hz than at 30 Hz.

The vibrotactile localization was achieved through a series of steps from the physical level to the perceptual level, including propagation of vibration from the vibration source, sensing of the skin deformation at mechanoreceptor level, and information processing in the brain. We believe that the factor of the difference between frequencies obtained in our experiment occurred somewhere in the above steps. Although it is not clear from the results of our experiment which step and which factor caused the difference, here we discuss the possible factors by introducing the results of previous studies.

One possible factor that influenced our experimental results at the physical level is the spread or attenuation of vibrations. When the vibration is applied to the skin of the hand, how it spreads depends on the vibration frequency (Manfredi et al., 2012; Reardon et al., 2019; Shah et al., 2019). For example, the attenuation was found to be smaller at higher frequencies where the peak response of the PC channel was at (200–250 Hz) (Manfredi et al., 2012; Reardon et al., 2019). This difference in the physical level may have influenced our experimental results, for example, the difference in the standard deviation of bias depending on stimulus frequency. The reason why the standard deviation difference was only observed when the stimulus was applied from a place distant from the subject's hand in our experiment might be as follows. First, the larger the distance is, the greater is the attenuation by the silicone rubber sheet. This leads to the gradient of intensity on skin being less and this makes the recognition of spatial intensity distribution difficult, which in turn results in a larger standard deviation. Also, there is a possibility that the vibration reaches a wider area when stimulating from a distant place rather than a near place and this might be related to the larger standard deviation.

There are several possible factors at the level of perception. For example, the characteristics of the receptors may be relevant. Previous studies have reported differences in the receptive field size (Schady and Torebjörk, 1983; Vallbo and Johansson, 1984) and the perceived size (Békésy, 1958) depending on the vibration frequencies. Also, differences depending on the frequency of the stimulus have been investigated in the study of phantom sensation, which has something in common with our experimental situation. Alles (1970) investigated the localization of phantom sensation using 30 and 100 Hz vibrations, and concluded that 100 Hz was more desirable because of humans' faster localization judgment at 100 Hz (but see also Bekesy, 1967). This may be related to our result that the standard deviation of bias was smaller at 230 Hz than at 30 Hz when the vibration source was far from the subject's hand.

There is a study that has looked at the capability of vibration localization at two different parts of the hand using a vibration stimulus of low frequency or high frequency. Sherrick et al.'s (1990) task was to discriminate the stimulus sites that vibrated at 25 or 250 Hz on the palm of the hand and they obtained results related to our results with respect to dependence on stimulus direction. Specifically, the discrimination results did not differ between 25 and 250 Hz on the little finger side of the palm in their experiment. This is consistent with our result that there is no significant difference in standard deviation between frequencies in 0° direction. In contrast, in Sherrick et al.'s experiment, the discrimination performance of 250 Hz was worse than that of 25 Hz in the area close to the wrist on the little finger side of the palm (hypothenar eminence). This is inconsistent with our results that a smaller standard deviation was observed for 230 Hz at 270 and 315°. The reason for this discrepancy may be the difference in the experimental apparatus (i.e., they used piezoceramic Bimorphs) or in the task.

### 4.5. Application Using Sinusoidal Vibration Localization

#### 4.5.1. Direction Information Presentation

Our results show that the method of presenting sinusoidal vibrations outside the body is promising for applications that present direction, while not suitable for applications that present distance. So if we apply our method to an application that warns the user of an approaching obstacle, for example, the user can recognize the directional information of the obstacle, but not the distance of the obstacle. As for the distance of the obstacle, it might be better to use other rule-based expressions (e.g., expressing distance information in terms of the intensity of the vibration, with a rule that a strong vibration represents a small distance and a weak vibration represents a large distance).

**Figure 9:**
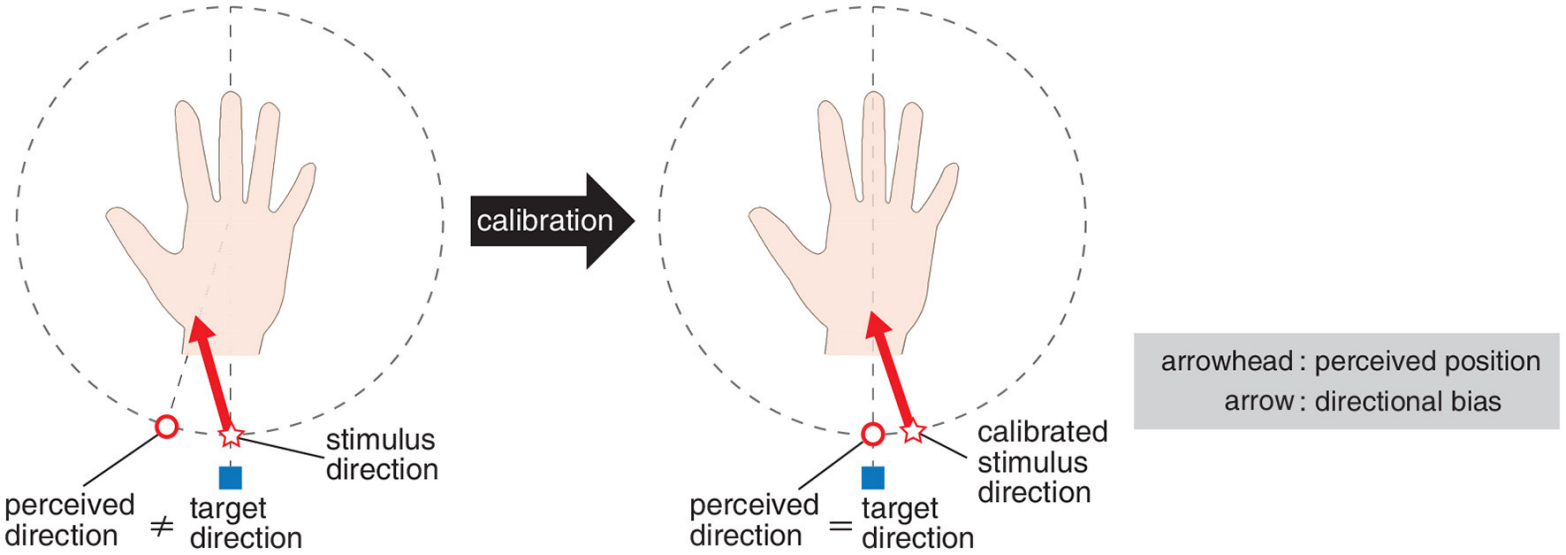
Calibration of stimulus direction in order to make the user recognize the target direction accurately.

In directional presentation applications, there would be a requirement to present users with a target direction accurately. We found that there is a bias both when 30 Hz and when 230 Hz stimuli were used, and the bias varied depending on the stimulus direction. Therefore, it might be effective to calibrate the actual stimulus direction according to the target direction in order to make the user recognize the target direction accurately (see example of calibration in Figure 9). An example of implementation is to prepare a lookup table that specifies the stimulus direction for each target direction and present the stimulus from the calibrated direction. (e.g., to make the user feel the stimulation in the center of the wrist, vibrate from the right side of the wrist.) There remain some issues in realizing this calibration: first, to make users recognize the target direction by presenting from the actual stimulus direction slightly different from the target one, the vibrotactile array should be arranged densely. For example, if the vibrotactile array is arranged sparsely, as in the stimuli layout used in this experiment, it may not be possible to provide stimulation from the calibrated direction that should be presented. In this respect, there is room for further consideration (e.g., utilization of phantom sensation). Second, the bias may vary depending on the device configuration (e.g., the material used as a medium) and the posture of the user's fingers, hands, and arms. It is known that the position of the perceived stimulus changes depending on the user's posture (Yamamoto and Kitazawa, 2001; Sadibolova et al., 2018). It would be necessary to evaluate the bias beforehand whenever the conditions, such as device or users' posture, change.

Another requirement that is related to the directional presentation application is to make users recognize multiple directional information. Specifically, we assume the case when the users need to recognize and discriminate between obstacles with different characteristics (e.g., cars and pedestrians) that are present in different directions. The requirements for such cases would be realized by representing the direction of different objects with different frequencies. In a previous study, it is known that when high and low frequencies of 200 ms duration are presented to fingertips at a 1 s interval, the two frequencies can be discriminated (Kuroki et al., 2013). A multiple direction presentation would be important for some applications. For example, in obstacle warning applications it is better to inform users of the presence of multiple obstacles around users and the locations of those obstacles. In the future we would like to investigate the feasibility of multiple direction presentation with the method using localization of sinusoidal vibrations with different frequencies.

From the viewpoint of a vibration presentation device that realizes a directional presentation application, there is a requirement to relax the layout constraints of vibrotactile actuators. Our results suggest that when stimulating from a distance of at least 180 mm, it would be better to use 230 Hz frequency vibration, at which frequency the standard deviation is <30 Hz. This policy of using 230 Hz vibrations can be regarded as a good choice considering the current distribution of vibration presentation devices. There are only a few commercially available vibration devices such as TacHammer that can present a strong amplitude at 30 Hz. In contrast, there are more devices that can output 230 Hz which are relatively available and easy to purchase (e.g., Haptuator and Haptic Reactor, which are often used in the tactile research field). If for some reason, we want to use 30 Hz vibrations instead of 230 Hz to stimulate from a distance, it will be no problem, as long as we can tolerate the relatively large standard deviation in the use cases.

#### 4.5.2. Position Information Presentation on Skin

Our finding that the vibration source is localized inside the hand area even when stimulated from outside the hand can be interpreted as showing that the physical position of the vibrotactile actuator and the perceived position of the vibration can be widely separated. In other words, it may be possible to use the separation to present information by vibration to the position inside the hand without placing a vibration source inside the hand. Although the conventional method that placed vibrotactile array in contact with a part of the user's body can present positional information inside the hand by directly stimulating the hand skin, the separation of stimulus and perception has the following advantages that may be useful in some situations.

One of the advantages is that it can reduce layout constraints. If we want to present stimulation at the hand position, usually, we need to place the vibrotactile actuators in the contact area of the hand. In addition, if we want to present the vibration in a distributed manner, we need to lay out multiple vibrotactile actuators in a limited space in contact with the hand. This integration of actuators is expensive to design. Therefore, if the vibrotactile actuators can be placed in a remote location, the layout constraints will be reduced.

Another advantage is that the tactile feel of the surface of the vibration presentation system where the user's hand touches could be improved. If there is a presentation system that includes a vibrotactile actuator right under the palm of the hand, the heat generated by the system may cause the tactile sensation to be poor. This can be solved by placing the presentation system containing the vibrotactile actuator remotely.

However, as shown in Figure 3, when the vibration source is placed remotely, the perceived position is limited and near the edge of the hand. This is useful for cases where we want users to localize the stimulus at the edge of the hand, but note that it is difficult to localize the stimulus near the center of the hand.

### 4.6. Limitations

The result of our experiment might be affected by the physical factors due to vibration source, medium, and the human body in our experimental settings.

In our experiment, the participants were instructed to place their hands naturally on the silicon sheet. This is because placing their hands naturally does not burden the user if the method is used as a spatial information presentation device. On the other hand, if the force that presses the hand against the silicon sheet is unnaturally large or small, the vibration propagated to the hand changes, and thus the recognition result might change.

Also, in future work, we will investigate how the localization accuracy changes when we change the material of the medium. In our experimental environment, the propagated vibration was not a clear sinusoidal waveform with a single frequency and attenuated to some extent. When we change the material of the medium, the characteristics of the propagated vibration will be different, which will affect the localization result. Indeed, a previous study shows that localization results changed with the material in the case of a one-dimensional medium due to a change in propagated vibration characteristics (Gongora et al., 2017).

## 5. Conclusion

In this study, we investigated the effectiveness of a method to cause users to localize a sinusoidal vibration source around the hand. Specifically, we conducted an experiment to investigate the localization ability when the source vibration was a low-frequency vibration (30 Hz) or a high-frequency vibration (230 Hz). The results of the experiment showed that it is difficult for subjects to recognize the distance of the vibration source but that the direction can be recognized relatively accurately. The perceived direction is relatively accurate in the sense that the perceived direction is very close to the true stimulus direction as shown in Figure 4A while the perceived distance bears little relation to the true distance and is almost constant and close to zero as shown in Figure 4B. Observed bias in the recognized direction depended on the vibration frequency. Furthermore, the standard deviation of the bias was smaller for high-frequency vibration than for low-frequency vibration as the stimulus distance increased. This indicates that it is possible to present directional information with high accuracy and small variability, as long as the appropriate frequency of vibration is selected according to the application and the position of the stimulus is properly calibrated.

## Data Availability Statement

The original contributions presented in the study are included in the article/supplementary material, further inquiries can be directed to the corresponding authors.

## Ethics Statement

The studies involving human subjects were reviewed and approved by Nippon Telegraph and Telephone Corporation (Approval number: R02-015 by NTT Communication Science Laboratories Ethics Committee). The subjects provided their written informed consent to participate in this study.

## Author Contributions

YU made the apparatus and conducted the experiments. YU and SK designed the experiments, analyzed the data, and authored the manuscript. All authors contributed to the article and approved the submitted version.

## Conflict of Interest

The authors declare that the research was conducted in the absence of any commercial or financial relationships that could be construed as a potential conflict of interest.

## Publisher's Note

All claims expressed in this article are solely those of the authors and do not necessarily represent those of their affiliated organizations, or those of the publisher, the editors and the reviewers. Any product that may be evaluated in this article, or claim that may be made by its manufacturer, is not guaranteed or endorsed by the publisher.
