# Supplementary material for: Sinusoidal Vibration Source Localization in Two-Dimensional Space Around the Hand
Source: Front Psychol. 2022 Jun 10;13:878397. doi: 10.3389/fpsyg.2022.878397 (PMC9232185; doi:10.3389/fpsyg.2022.878397)
Supplement: Supplementary file 1 [file Data_Sheet_1.PDF]

Supplementary Material

Supplementary Figure 1. Experimental Procedure. The experiment was composed of a familiarization phase and a test phase.

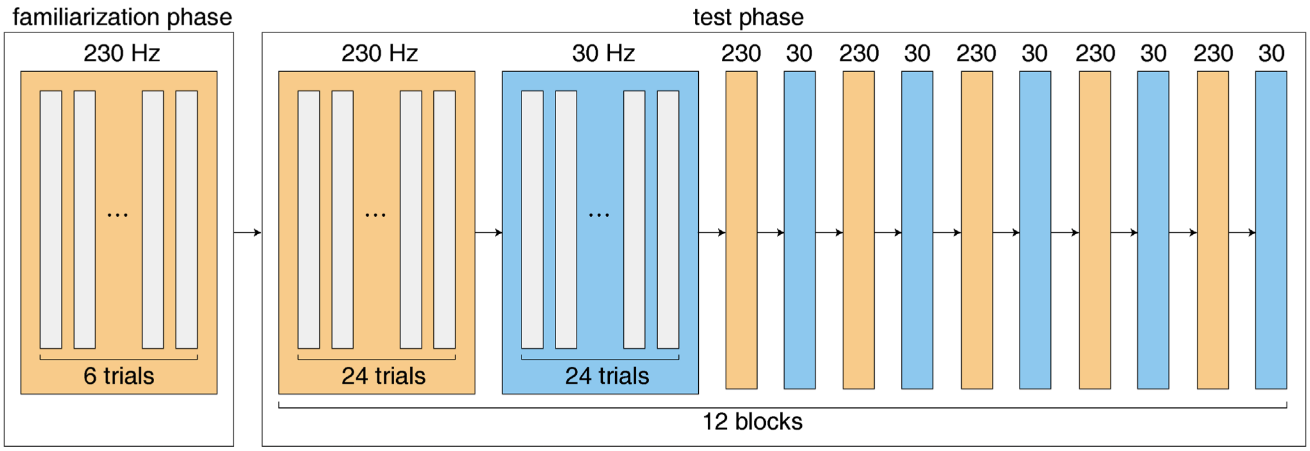

Supplementary Table 1. 3-way ANOVA results of the directional bias

	F	Df	p
stimulus direction	16.51	7	0.0001
stimulus distance	13.39	2	0.250
frequency	0.17	1	0.684
stimulus direction * stimulus distance	1.00	14	0.449
stimulus direction * frequency	4.36	7	0.0001
stimulus distance * frequency	1.85	2	0.159
stimulus direction * stimulus distance * frequency	1.19	14	0.276

Supplementary Table 2. 3-way ANOVA results of the standard deviation of directional bias.

	F	Df	p
stimulus direction	11.51	7	0.0001
stimulus distance	55.16	2	0.0001
frequency	61.19	1	0.0001
stimulus direction * stimulus distance	1.94	14	0.021
stimulus direction * frequency	5.76	7	0.0001
stimulus distance * frequency	27.47	2	0.0001
stimulus direction * stimulus distance * frequency	2.19	14	0.008
